# *In Vitro* Evaluation of the Antibacterial Activity of EndoSeal MTA, iRoot SP, and AH Plus against Planktonic Bacteria

**DOI:** 10.3390/ma15062012

**Published:** 2022-03-08

**Authors:** Siew Thong Mak, Xin Fang Leong, In Meei Tew, Endang Kumolosasi, Lishen Wong

**Affiliations:** 1 Department of Restorative Dentistry, Faculty of Dentistry, Universiti Kebangsaan Malaysia, Kuala Lumpur 50300, Malaysia; siewthong1103@gmail.com (S.T.M.); inmeei@ukm.edu.my (I.M.T.); 2 Department of Craniofacial Diagnostics and Biosciences, Faculty of Dentistry, Universiti Kebangsaan Malaysia, Kuala Lumpur 50300, Malaysia; leongxinfang@ukm.edu.my; 3 Drug and Herbal Research Centre, Faculty of Pharmacy, Universiti Kebangsaan Malaysia, Kuala Lumpur 50300, Malaysia; e_kumolosasi@ukm.edu.my

**Keywords:** antibacterial, modified direct contact test, endodontic sealer, bacteria, planktonic

## Abstract

This study aimed to investigate the antibacterial activity of three endodontic sealers, AH Plus, iRoot SP, and EndoSeal MTA, against four planktonic bacteria species. The antibacterial activity of the three endodontic sealers was assessed using a modified direct contact test. Bacteria suspension of *Actinomycoses viscosus, Enterococcus faecalis*, *Staphylococcus aureus*, and *Streptococcus mutans* were left in contact with the sealers that were pre-set or set for 1, 3, 7, and l4 days for an hour. Freshly mixed AH Plus and EndoSeal MTA were highly effective against all four tested bacteria as no surviving bacteria were recovered after treatment. Meanwhile, freshly mixed iRoot SP was not able to kill all bacteria, regardless of the species, demonstrating a weak antibacterial effect. After 24 h, AH Plus lost its antibacterial activity. EndoSeal MTA showed a strong and extended bactericidal effect against *S. aureus* and *S. mutans* for 3 days and *A. viscosus* for 7 days. In conclusion, fresh AH Plus and EndoSeal MTA exhibited a potent effect against all four bacteria species. EndoSeal MTA remained effective after setting when tested against *A. viscosus, S. aureus*, and *S. mutans*. Among all tested sealers, iRoot SP demonstrates the weakest antibacterial activity.

## 1. Introduction

Complete elimination of microorganisms residing within the infected root canal system has always been the ultimate goal of endodontic treatment [1]. The successful rate of endodontic treatment is mainly composed of three main stages, including the removal of infected pulp tissues within the tooth [2,3,4], disinfection and shaping of the root canals, and lastly, the filling of the root canal chamber with inert materials [5,6,7,8]. Although each stage is equally important, the success rate of endodontic treatment is heavily dependent on the final stage [9]. Two main components are involved in the root canal filling phase, including a solid core material and a sealer [5]. The solid core material fills most of the space within the root canal while the endodontic sealer fills the remaining space, especially those within the accessory canals [10].

While gutta-percha has been the most common core material being used [11], there are quite several endodontic sealers available in the market. According to Komabayashi et al. [12], there is a total of eight endodontic sealer types. Among these endodontic sealer types, tricalcium silicate-based endodontic sealers have gained popularity over the years due to their benefits, such as biocompatibility, lower microleakage, and antimicrobial activity. Despite that many previous studies were carried out to investigate the antimicrobial activity of various endodontic sealers [13,14,15,16,17,18], little information is available regarding the antibacterial activity of the two relatively new tricalcium silicate-based endodontic sealers in the market, namely iRoot SP (Innovative BioCeramix Inc., Vancouver, BC, Canada), and EndoSeal MTA (Maruchi, Wonju, South Korea) [14,19].

*Enterococcus faecalis* was frequently isolated from endodontic infections (with a prevalence ranging from 45.8% to 89.6%) [20,21,22] and was always associated with endodontic failure [23]. It was also reported that the resistance of *E. faecalis* was mainly due to its ability to survive in harsh environments and develop antibiotic resistance, as well as the capability of forming biofilm [23]. Thus, this microorganism has been widely used in various endodontic studies for studying the antimicrobial properties of different disinfecting agents or dental materials [13,14,24,25,26,27]. Nevertheless, most studies did not include other bacterial species, which have also been reported to be associated with endodontic infections. For instance, the association of *Streptococcus mutans* with endodontic infections was also proven in various studies [28,29,30], whereby it is commonly recovered (70%) from infected root canals [31]. Its ability to survive in the root canal is similar to those of *E. faecalis,* including being able to endure adverse environments and form biofilms [32]. In addition, *Staphylococcus* spp. and *Actinomyces* spp. were also frequently isolated from teeth samples associated with endodontic failure [33,34,35,36], with their prevalence ranging from 2.75% to 16.35% and 12.5% to 52%, respectively. The persistence of *S. aureus* is due to its antimicrobial resistance and ability to produce exotoxins which aid in the colonization within the host [37]. Meanwhile, *A. viscosus* is persistent mainly due to its ability to form biofilm. Hence, it is important to include bacterial species other than *E. faecalis* in endodontic studies.

Agar diffusion test (ADT) was the most common method in the past for investigating antimicrobial properties of endodontic sealers [38,39,40,41]. However, this method is not recommended nowadays due to its well-known limitations [42] since it often does not reflect the true antimicrobial activity of tested sealers or disinfecting agents. A method known as the direct contact test (DCT) was then introduced by Weiss et al. [24] to overcome the disadvantages of ADT. The DCT is a reproducible and quantitative method that allows the assessment of endodontic sealers, which are mostly insoluble.

This study aimed to investigate the antibacterial activity of three endodontic sealers, including AH Plus, iRoot SP, and EndoSeal MTA, using a modified DCT. Four planktonic bacterial species were chosen to be tested against the selected endodontic sealers, including *A. viscosus*, *E. faecalis*, *S. aureus*, and *S. mutans.*

## 2. Materials and Methods

### 2.1. Endodontic Sealers

Three types of endodontic sealers were used, including AH Plus (Dentsply DeTrey, Konstanz, Germany), iRoot SP (Innovative BioCeramix Inc., Vancouver, BC, Canada), and EndoSeal MTA (Maruchi, Wonju, South Korea). All three endodontic sealers were prepared according to the instructions provided by their manufacturer before being used. For AH Plus, two different tubes containing paste A and paste B individually were included in the package. Equal volume units (1:1) of paste A and paste B of AH Plus were mixed evenly with a metal spatula on the mixing pad supplied along with the package. The mixed paste was mixed to a homogenous consistency before being used. Meanwhile, both iRoot SP and EndoSeal MTA were available in a convenient premixed ready-to-use form, both packaged in a preloaded syringe. They were applied directly after replacing the cap with the needle tips included along with the package.

### 2.2. Bacteria

Four strains of bacteria were used in this study, including *Enterococcus faecalis* American Type Cell Culture Collection (ATCC) 29212 (ATCC, Rockville, MD, USA), *Streptococcus mutans* ATCC 700610 (ATCC, Rockville, MD, USA), *Staphylococcus aureus* ATCC 25923 (ATCC, Rockville, MD, USA), and *Actinomyces viscosus* ATCC 15987 (ATCC, Rockville, MD, USA).

### 2.3. Bacteria Suspension Preparation

*E. faecalis*, *S. aureus*, and *S. mutans* were grown overnight for 18 h at 37 °C, while *A. viscosus* was grown for 72 h at 37 °C according to the ATCC product sheet. Both *E. faecalis* and *S. aureus* were grown aerobically while *A. viscosus* and *S. mutans* were grown in a 5% CO_2_ supplemented atmosphere. Additionally, Brain Heart Infusion Agar/Broth (Oxoid, Hampshire, UK) was used to grow all strains of bacteria except for *S. aureus*. Trypticase Soy Agar/Broth (Oxoid, Hampshire, United Kingdom) was used for growing *S. aureus.*

The bacteria suspensions were centrifuged at 5000× *g* for 5 min at room temperature before being resuspended in Phosphate Buffered Saline (PBS) to an optical density at 600 nm (OD_600_) of 1.0, corresponding to approximately 2 × 10^8^ colony-forming units (CFU)/mL for the modified direct contact test (MDCT) assay.

### 2.4. MDCT Antibacterial Assay

The MDCT was first introduced by Zhang et al. [14] for the investigation of the antibacterial activity of endodontic sealers. Firstly, each sealer was prepared beforehand, and approximately 0.5 mL was coated on the side wells of the 96-well microtiter plate held vertically by using a small-size round-ended dental instrument. Then, the MDCT was conducted individually for all four bacteria strains. Each endodontic sealer was used freshly mixed or after 24 h, 3 days, 7 days, and 14 days stored in 100% humidity at 37 °C.

**Figure 1:**
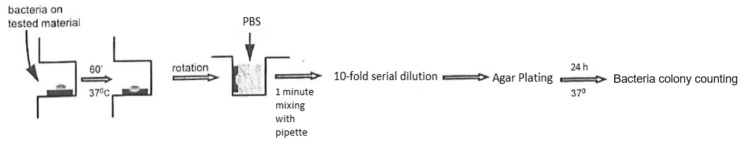
Schematic representation of MDCT.

An amount of 10 μL of each bacteria suspension (approximately 2 × 10^6^ CFU/mL) was carefully placed on the surface of each endodontic sealer. While the plate remained in the vertical position, wells were inspected for evaporation of the suspension’s liquid, which occurred within 1 h at 37 °C. Subsequently, 300 μL of PBS was added to each well. After gently mixing with a pipette for 1 min, 10-fold serial dilutions were performed by using PBS. The survival of bacteria was assessed by culturing the aliquots of 100 μL onto respective agar plates after 10-fold serial dilutions. After incubation for 24 h (except for *A. viscosus,* which needed 72 h) at 37 °C, colonies on the agar plates were counted, and the CFU/mL was calculated. Experiments were conducted in triplicate. A schematic figure of MDCT (Figure 1) is included below.

### 2.5. Statistical Analysis

The collected data were analyzed by using SPSS IBM statistical software version 25.0 (SPSS, Chicago, IL, USA). The difference between surviving bacteria after treatment and the negative control was analyzed with a one-way analysis of variance (ANOVA), followed by Tukey’s post-hoc test for multiple comparisons with the level of significance set at *p* < 0.05.

## 3. Results

**Figure 2:**
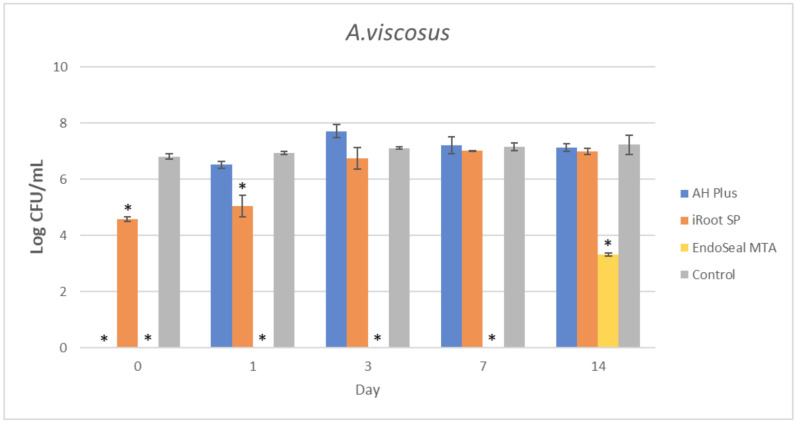
Mean log CFU/mL of *A. viscosus* in planktonic form after direct contact with AH Plus, iRoot SP, and EndoSeal MTA. Sealers were tested after freshly mixed, 1 day, 3 days, 7 days, and 14 days. * indicates statistically significant differences between each endodontic sealer and the negative control, *p* < 0.001.

**Figure 3:**
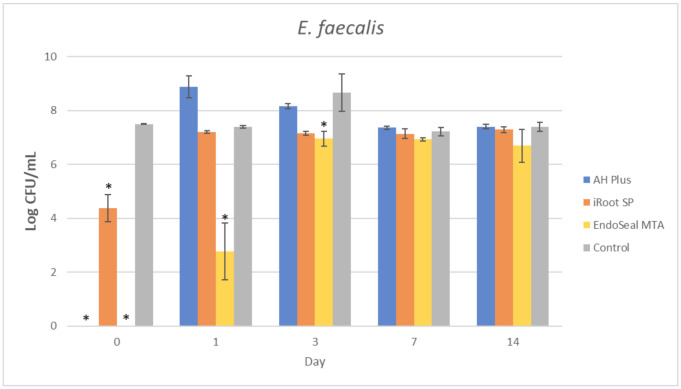
Mean log CFU/mL of *E. faecalis* in planktonic form after direct contact with AH Plus, iRoot SP, and EndoSeal MTA. Sealers were tested after freshly mixed, 1 day, 3 days, 7 days, and 14 days. * indicates statistically significant differences between each endodontic sealer and the negative control, *p* < 0.001.

**Figure 4:**
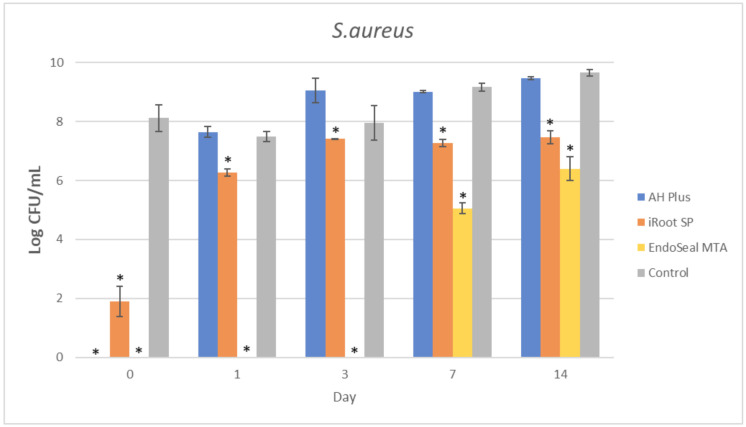
Mean log CFU/mL of *S. aureus* in planktonic form after direct contact with AH Plus, iRoot SP, and EndoSeal MTA. Sealers were tested after freshly mixed, 1 day, 3 days, 7 days, and 14 days. * indicates statistically significant differences between each endodontic sealer and the negative control, *p* < 0.001.

**Figure 5:**
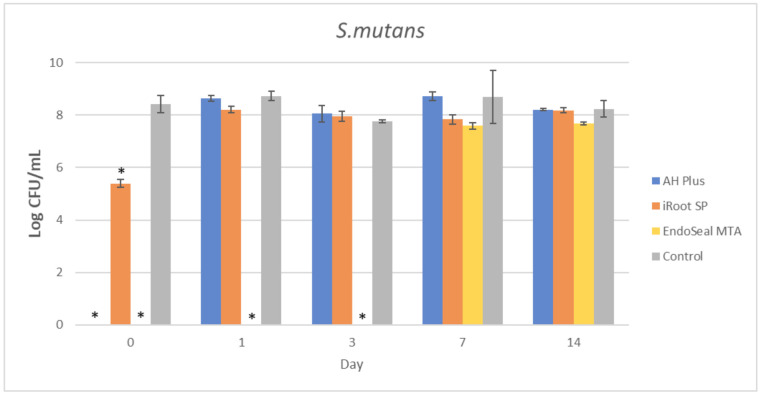
Mean log CFU/mL of *S. mutans* in planktonic form after direct contact with AH Plus, iRoot SP, and EndoSeal MTA. Sealers were tested after freshly mixed, 1 day, 3 days, 7 days, and 14 days. * indicates statistically significant differences between each endodontic sealer and the negative control, *p* < 0.001.

The mean amount of surviving planktonic *A. viscosus* after being treated with different ages of AH Plus, iRoot SP, and EndoSeal MTA are compared and illustrated in Figure 2. As for the MDCT results of *E. faecalis*, *S. aureus*, and *S. mutans*, they are illustrated in Figure 3, Figure 4 and Figure 5, respectively.

It could be observed that there were no surviving bacteria for all four bacterial species when treated with freshly mixed AH Plus, indicating a strong and effective antibacterial activity. Nevertheless, the antibacterial activity of AH Plus was lost after 24 h of setting, regardless of bacterial species.

The bioceramic sealer, iRoot SP, also demonstrated antibacterial activity against all four bacterial species when freshly mixed. However, its antibacterial activity is weaker as compared to the AH Plus since surviving bacteria could still be recovered. Among the four tested bacterial species, *S. aureus* was more susceptible to iRoot SP. In Figure 4, it could be observed that the iRoot SP showed a weak and extended antibacterial activity against *S. aureus* up to 14 days after setting (*p* < 0.001).

Meanwhile, EndoSeal MTA has exhibited the strongest antibacterial activity among the three endodontic sealers. The freshly mixed EndoSeal MTA sealer demonstrates strong antibacterial activity against all four bacterial species as there was a significant difference between the number of bacteria being recovered compared with the negative control (*p* < 0.001). EndoSeal MTA also demonstrated extended antibacterial activity against *S. aureus,* and *S. mutans* up to 3 days after setting. Besides, it was shown to be effective against *A. viscosus* even after 14 days of setting.

## 4. Discussion

Ideally, endodontic sealers should be dimensionally stable and non-toxic; they should be able to create a strong bond with the root canal dentin to seal well and prevent microleakage [5]. It is also favorable if the endodontic sealers exhibit strong, long-lasting antimicrobial effects and therapeutic effects [43]. Additional antimicrobial effects of the endodontic sealer would be beneficial in eliminating residual microorganisms, which have survived both chemical and mechanical instrumentation in endodontic therapy. As a result, the success rate of modern endodontic therapy can be increased.

Currently, there is a wide variety of endodontic sealers available commercially, and most of them have been studied thoroughly ever since being introduced. Endodontic sealers that have been studied widely include Sealapex, Epiphany, GuttaFlow, RoekoSeal, Tubli Seal, and Endosequence [14,27,40,44,45]. Despite that a large number of studies investigated the antibacterial effect of various endodontic sealers in the past, relatively few amounts of studies have investigated iRoot SP [14,46,47,48] and EndoSeal MTA [15,49,50]. This is because these two endodontic sealers are relatively new in the market, especially the EndoSeal MTA, which was introduced around the year 2014 [51]. As for iRoot SP, most studies researched on EndoSequence BC, which is a similar sealer but marketed under different brand names [12]. Hence, the investigation of the antibacterial activity of these two endodontic sealers (iRoot SP and EndoSeal MTA) would be the focus of this study.

As mentioned earlier, the MDCT method used in this study is reproducible and quantitative. As compared to the traditional DCT, this modified version has managed to retain its advantages and improve some of its disadvantages. For example, the MDCT allows the measuring of the bactericidal effect instead of the bacteriostatic effect of the tested endodontic materials [14], which is important in endodontic clinical practice. Another important advantage of MDCT is that the results obtained will not be affected by the nature of endodontic sealers easily. There is a high possibility for endodontic sealers that set slower to affect the results reading. Since unset sealers turn the broth cloudy in the well during the mixing procedure, it will then affect the absorption of light and, ultimately, the reading of the spectrophotometer [52]. Whereas in MDCT, the results are collected through bacteria culturing on an agar medium, and quantification of surviving bacteria is also possible.

*E. faecalis*, *S. aureus*, and *A. viscosus* were all reported to be associated with post endodontic treatment infection [53,54,55]. These bacteria can survive from chemical irrigation or reinfect the root canal through microleakage. One of the main factors that favor the growth of these three bacterial species within the root canal system would be their ability to attach to dental surfaces, which leads to the formation of biofilm. In addition, *E. faecalis* was reported to be more superior than other bacteria species on account of its ability to attach to collagen within dentinal tubules even in an adverse environment [55]. *S. mutans* was also isolated in cases of endodontic re-infection [55]; therefore, it was chosen to be tested in the current study.

In the present study, the AH Plus was also included for comparison of antibacterial activity with the other two tested endodontic sealers. This is because the antibacterial effect of AH Plus was well established in previous studies [14,44,56]; thus, it was selected due to its predictable pattern in antibacterial activity. As shown in Figure 2, Figure 3, Figure 4 and Figure 5, fresh AH Plus demonstrates a strong antibacterial effect. The fresh AH Plus is potent since no surviving bacteria were recovered, regardless of the bacteria species. Despite that, the antibacterial activity of AH Plus was lost after 24 h. This trend is similar for all four bacterial species, whereby no antibacterial activity could be observed when the sealer was tested after being aged for 1 day, 3 days, 7 days, and 14 days. Zhang et al. [14] also reported similar results for *E. faecalis*, while Kapralos et al. [13] reported similar results for *E. faecalis*, *S. mutans*, and *S. aureus*. Previous endodontic studies using DCT and ADT have also reported similar results [40,56,57,58], regardless of the investigated bacterial species.

It was widely recognized that the bactericidal effect of AH Plus is due to the release of formaldehyde, which occurs during the polymerization process [44,59,60]. However, AH Plus was improved from AH 26, an endodontic sealer that releases a higher amount of formaldehyde that may cause genotoxicity and cytotoxicity. Hence, the manufacturer improved the AH Plus to minimize the discharge of formaldehyde. It was reported that there is a possible association between the bactericidal effect of resin-based sealers with epoxy-derived bisphenol-A-diglycidyl-ether [61]. Schweikl and Schmalz [62] also reported that both epoxy resin from paste A and amines from paste B of the AH Plus could reduce cell viability, suggesting that the toxic effect of unpolymerized components is the reason for its potent antibacterial effect. The greatly diminished antibacterial effect of the set AH Plus may be due to the polymerization process which depletes the epoxy resin and amines.

The iRoot SP is a relatively new endodontic sealer, unlike the AH Plus. This sealer is mainly based on tricalcium silicate, as shown in Table 1, well known for its biocompatibility since it has no cytotoxicity [12,63]. The present study shows that fresh iRoot SP possessed a weak antibacterial activity, which is not in accordance with most studies in the past, which have reported a strong antibacterial activity of MTA-type materials. Zhang et al. [14] reported that such sealer exhibited a potent antibacterial effect up to 3 days against *E. faecalis*. Meanwhile, Kapralos et al. [13] also reported the potent antibacterial effect of TotalFill BC sealer (which is similar to iRoot SP but marketed under different brand names) against *E. faecalis*, *S. mutans*, *S. epidermis*, and *S. aureus* up to 7 days. Nevertheless, it was reported in a review conducted by Parirokh and Torabinejad [64] that previous studies evaluating the antimicrobial effect of MTA materials were confounding and produced contradictory results.

Most studies reported that the bactericidal effect of MTA sealers exhibited through increasing local pH, which is caused by a hydration cycle of calcium silicate [13,14,65]. The calcium silicates composition of the iRoot SP sealer should be able to release calcium and hydroxide ions by utilizing the moisture available in the root canal. Meanwhile, calcium hydroxide is also produced and reacts with phosphate to form water [66], which plays a role in activating the cycle again. Therefore, there is a possibility that lack of moisture in the experiment setup of the current study might be the reason for iRoot SP to show a weak antibacterial activity.

Another possibility might be due to the chemical composition of iRoot SP. It was reported by Shin and his colleagues [67] that iRoot SP has weaker effects due to a lower amount of oxide compounds with antimicrobial effect, specifically those with the ability to destroy cell walls of Gram-positive bacteria. This is especially important as the cell wall of Gram-positive bacteria can decrease the penetration of calcium hydroxide into the bacteria cells, whereby the calcium hydroxide aids in the denaturation of bacteria DNA and protein [67]. In the present study, all four bacterial species being tested were known to be Gram-positive bacteria. Thus, this may explain the lower antibacterial activity of iRoot SP as compared with the other two endodontic sealers.

However, this does not explain the weak antibacterial activity of iRoot SP against *S. aureus,* as mentioned above. This finding is comparable with the results of Kapralos et al. [13]. In their study, it was suggested that the presence of moisture might reduce the bactericidal effect of TotalFill BC sealer against *S. aureus*. In the present study, since no additional water was added to the sealers during incubation, this may explain the weak antibacterial effect of iRoot SP against *S. aureus* since it is typically hydrophobic [68]. In the presence of water, the diffusion of compounds with antibacterial activity from the sealer into *S. aureus* will be interrupted due to the disrupting attachment of bacterial cells onto the iRoot SP sealer.

EndoSeal MTA was not introduced until recently; hence, studies exploring its antimicrobial activity are limited [49,50,67]. The previous antibacterial assessments of EndoSeal MTA were performed using ADT, thus making this study the first to assess its antibacterial activity with MDCT. The main composition of EndoSeal MTA is similar to iRoot SP, suggesting that the high antibacterial activity of EndoSeal MTA can be due to a combination of high pH and active calcium hydroxide diffusion. What differentiates EndoSeal MTA from iRoot SP is that the former contains higher amounts of oxide compounds with antimicrobial activity than the latter, for example, sodium oxide, magnesium oxide, aluminum oxide, sulfur dioxide, and ferric oxide [67]. These oxide compounds are capable of damaging the bacteria cell wall, enhancing the permeability of molecules such as calcium hydroxide into the bacteria cell cytoplasm. Thus, explaining the most potent antibacterial effect of EndoSeal MTA among the sealers being tested. Although previous studies reported a strong bactericidal effect of EndoSeal MTA against *E. faecalis* [49,67], they did not include set/aged endodontic sealers. Since the depletion of calcium hydroxide in iRoot SP causes the decrease in antibacterial effect over time [14], it is also possible that there may be depletion in the number of oxide compounds or even calcium hydroxide, which lead to the reduced antibacterial activity of set/aged EndoSeal MTA sealer as reported in the present study. However, further research is required to investigate factors such as moisture, aging time, and bacteria species on the antibacterial activity of EndoSeal MTA. Nevertheless, it was proven in the present study that it is possible for endodontic sealers such as iRoot SP and EndoSeal MTA to exhibit prolonged antibacterial effect up to 14 days as compared to the previous studies.

Since EndoSeal MTA demonstrated a potent antibacterial effect across an extended time span, it could be recommended to dental practitioners. Dental practitioners may consider the application of EndoSeal MTA for patients with recurrent and/or persistent endodontic infection, especially those with a past medical history of antibiotic resistance. Mechanical cleaning of the canal system remains important for the elimination of intracanal bacteria loads [2,3,4]. Although all three investigated endodontic sealers exhibited a certain degree of antibacterial activity, none of them were able to demonstrate a strong and long-lasting antibacterial effect against all four planktonic bacteria. Besides, it was reported that antibacterial assays using planktonic bacteria do not represent endodontic infections in vivo. This is because bacteria cells are more likely to be found in an organized manner when attached to the root canal walls, which is known as a biofilm. Future research should be conducted to investigate the antibacterial activity of endodontic sealers utilizing a biofilm model, particularly multispecies biofilm. Another limitation is that no chemical investigation was performed to determine the compound released by the tested sealers, and hence the exact mechanism of action could not be validated. Additional investigations utilizing chromatography and/or spectroscopy should also be considered for the analysis of the chemical composition profile of each endodontic sealer to uncover their mechanism of action.

## 5. Conclusions

In conclusion, all three endodontic sealers investigated in the present study exhibited various degrees of antibacterial activity against *A. viscosus, E. faecalis, S. aureus*, and *S. mutans* when tested before setting. Aged iRoot SP had extended antibacterial activity up to 14 days against *S. aureus*, while EndoSeal MTA had extended antibacterial activity against *A. viscosus* and *S. aureus* for up to 14 days. This study had also indicated that EndoSeal MTA demonstrated the strongest and elongated antibacterial activity.

## Figures and Tables

**Table 1:** Endodontic sealers and their composition.

Type	Product Name		Composition	References
Epoxy resin	AH Plus (Dentsply DeTrey, Konstanz, Germany)	Paste A	Bisphenol A epoxy resin, Zirconium oxide, Bisphenol F epoxy resin, Calcium tungstate, Iron oxide, Silica	Manufacturer Instruction Sheet
		Paste B	N, N-dibenzyl-5-oxanonadiamin-1,9, Amantiameamine, Tricyclodecane-diamine, Calcium tungstate, Zirconium oxide	
Tricalcium silicate (MTA/Bioceramic)	iRoot SP (Innovative Bioceramix Inc, Vancouver, Canada)	One paste	Zirconium oxide, Calcium silicates, Calcium phosphate, Calcium hydroxide, Filler, Thening agents	Manufacturer Instruction Sheet
	Endoseal MTA (Maruchi, Wonju, South Korea)	One paste	Calcium silicates, Calcium aluminates, Calcium sulfate, Radiopacifier, Thickening agent	Manufacturer Instruction Sheet

## Data Availability

Not applicable.
